# Supplementary material for: Synthesis and Vulcanization of Polymyrcene and Polyfarnesene Bio-Based Rubbers: Influence of the Chemical Structure over the Vulcanization Process and Mechanical Properties
Source: Polymers (Basel). 2022 Mar 30;14(7):1406. doi: 10.3390/polym14071406 (PMC9003078; doi:10.3390/polym14071406)
Supplement: Supplementary file 1 [file polymers-14-01406-s001.zip › polymers-1644524-supplementary.pdf]

Synthesis and Vulcanization of Polymyrcene and Polyfarnesene Bio-based Rubbers: Influence of the Chemical Structure over the Vulcanization Process and Mechanical Properties

Arnulfo Banda-Villanueva [1], José Luis González-Zapata [1], Manuel Eduardo Martínez- Cartagena [1], Ilse Magaña [1], Teresa Córdova [1], Ricardo López [1], Luis Valencia [2], Sergio García Medina [3], Alejandro Medina Rodríguez [3], Florentino Soriano [1] and Ramón Díaz de León [1,*]

1 Research Center for Applied Chemistry, Blvd Enrique Reyna 140, San José de los Cerritos, 25294, Saltillo, Co-ahuila, México; abanda.d18@ciqa.edu.mx (A.B.V.); gonzalezjl.d17@ciqa.edu.mx (J.L.G.Z.); eduardo-cartaa@gmail.com (M.E.M.C.); ilsma.rivera58@gmail.com (I.M.); trscordova@gmail.com (T.C.); ricar-do.lopez@ciqa.edu.mx (R.L.).

2 Biofiber Tech Sweden AB, Norrsken Hourse, Birger Jarlsgatan 57C, SE11356, Stockholm; luisalex_val@hotmail.com (L.V.).

3 CIATEC, Omega 1201, Colonia Industrial Delta, 37545. León, Guanajuato, México; smedina@ciatec.mx (S.G.M.); lmedina@ciatec.mx (A.M.R.).

* Correspondence: ramon.diazdeleon@ciqa.edu.mx (R.D.d.L.);

Table S1. Data obtained from rheometric curves in the crosslinking process of polydienes with the CV system.

Sample	Rubber	M_L (dNm)	M_H (dNm)	ΔM (dNm)	CRI (min^{-1})	t_{s2} (min)	t_{c90} (min)
1CV	PB-H	1.46	4.96	3.50	3.53	4.97	33.27
2CV	PB-H-CB[a]	8.14	11.48	3.34	4.08	2.38	26.85
3CV	PI-H	0.24	1.61	1.37	9.60	3.24	13.65
4CV	PI-H-CB[a]	2.44	10.59	8.15	7.21	0.96	14.82
5CV	PM-H	0.06	1.18	1.12	2.76	4.75	41.02
6CV	PM-H-CB[a]	0.67	5.11	4.44	6.42	0.72	16.28
7CV	PF-H	0.03	0.44	0.41	2.59	5.34	43.99
8CV	PF-H-CB[a]	0.77	3.77	3.00	5	1.50	21.50

9CV	PB-L	0.24	2.41	2.17	2.69	5.45	42.63
10CV	PB-L-CB[a]	1.48	9.95	8.47	2.43	1.39	42.55
11CV	PI-L	0.05	0.86	0.81	7.36	2.95	16.53
12CV	PI-L-CB[a]	2.03	9.67	7.64	8.86	1.10	12.39
13CV	PM-L	0.01	0.10	0.09	9.67	9.08	19.42
14CV	PM-L-CB[a]	0.78	3.72	2.94	4.20	1.51	25.29
15CV	PF-L	-	-	-		-	-
16CV	PF-L-CB[a]	0.23	1.51	1.28	3.97	1.85	27.04

[a] Carbon black reinforced compounds.

Table S2. Data obtained from rheometric curves in the crosslinking process of polydienes with the EV system.

**Sample**	**Rubber**	**M_L (dNm)**	**M_H (dNm)**	**ΔM (dNm)**	**CRI (min^{-1})**	**t_{s2} (min)**	**t_{c90} (min)**
1EV	PB-H	1.30	6.23	4.93	2.80	9.41	45.05
2EV	PB-H-CB[a]	7.49	20.45	12.96	9.68	4.15	14.48
3EV	PI-H	0.21	2.94	2.73	4.24	7.58	31.17
4EV	PI-H-CB[a]	2.20	13.20	11.00	16.69	0.83	6.82
5EV	PM-H	0.06	1.48	1.42	3.64	6.67	34.11
6EV	PM-H-CB[a]	1.70	8.29	6.59	13.86	0.70	7.91
7EV	PF-H	0.03	0.74	0.71	3.38	12.67	42.26
8EV	PF-H-CB[a]	0.86	4.68	3.82	15.50	1.85	8.30
9EV	PB-L	0.14	3.03	2.89	2.55	16.04	55.22
10EV	PB-L-CB[a]	1.78	13.99	12.21	7.48	0.86	14.22

11EV	PI-L	0.05	1.73	1.68	6.53	8.26	23.56
12EV	PI-L-CB[a]	1.54	9.65	8.11	14.47	0.49	7.40
13EV	PM-L	0.01	0.46	0.45	5.89	10.35	27.33
14EV	PM-L-CB[a]	0.53	4.53	4.00	13.37	1.75	9.23
15EV	PF-L	-	-	-	-	-	-
16EV	PF-L-CB[a]	0.27	2.83	2.56	17.18	2.07	7.89

[a] Carbon black reinforced compounds.

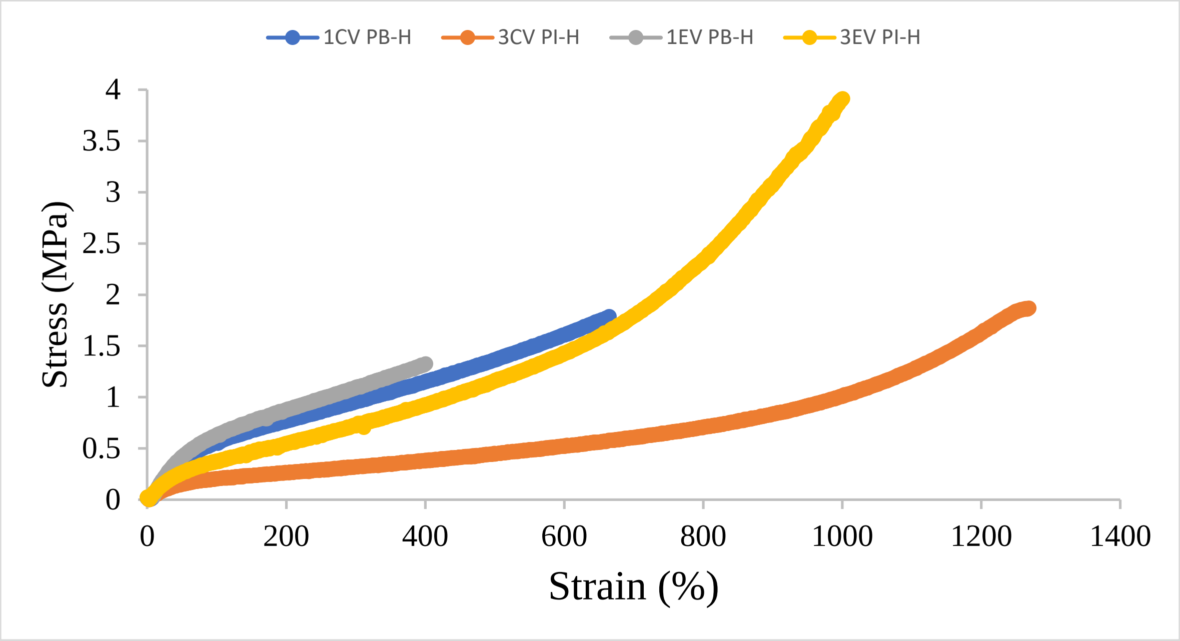

Figure S1. Exemplary stress-strain curves of some of the obtained composites..
